# Role of SPECT and SPECT/CT in the Surgical Treatment of Primary Hyperparathyroidism

**DOI:** 10.1155/2011/141593

**Published:** 2011-06-21

**Authors:** Michele L. Taubman, Melanie Goldfarb, John I. Lew

**Affiliations:** Division of Endocrine Surgery, DeWitt Daughtry Family Department of Surgery, University of Miami Leonard M. Miller School of Medicine, Miami, FL 33136, USA

## Abstract

Primary hyperparathyroidism is the most common cause of hypercalcemia in the outpatient population. This condition is usually the result of a single hyperfunctioning parathyroid gland. Targeted parathyroidectomy guided by intraoperative parathyroid hormone monitoring (IPM) through a small cervical incision has replaced traditional bilateral neck exploration (BNE) as the initial approach in the surgical treatment of primary hyperparathyroidism at many medical centers worldwide. Preoperative sestamibi-technetium 99m scintigraphy serves as an important prerequisite for successful targeted parathyroidectomy. Single-photon emission computed tomography (SPECT) and CT fusion, however, is a recent imaging technique that provides a three-dimensional functional image with advanced contrast resolution to greatly improve preoperative localization of parathyroid tumors.

## 1. Introduction

Primary hyperparathyroidism is the most common cause of hypercalcemia in the outpatient population. The delicate balance of calcium homeostasis in the human body has long been appreciated. Serum elevation of this mineral secondary to overproduction of parathyroid hormone is associated with fatigue, musculoskeletal pain, weakness, polyuria, nocturia, renal stones, memory loss, constipation, polydipsia, heartburn, and depression. At the present time, most patients with hyperparathyroidism do not present with symptoms of hypercalcemia, but rather are identified on routine biochemical screening. The underlying cause of primary hyperparathyroidism is usually a single parathyroid adenoma in 85–96% of cases while less frequent causes include double adenoma, parathyroid hyperplasia, and parathyroid carcinoma. Parathyroidectomy is indicated in all patients with symptomatic hyperparathyroidism and in those individuals with asymptomatic hyperparathyroidism that satisfies certain consensus criteria. These most recent guidelines include a serum calcium concentration >1.0 mg/dL above the upper limit of normal, creatinine clearance <60 mL/min, bone density >2.5 standard deviations below standard reference values for sex-matched peak bone mass at any site (*T*-score <-2.5), age <50 years old, inability or unwillingness to be followed, or a severe psychoneurologic disorder [1]. 

Since the first attempted parathyroidectomy in 1925 by Mandl, the surgical treatment for primary hyperparathyroidism has undergone a gradual evolution [2]. The large collar incision has been replaced by a very small 2–4-centimeter lower neck incision, and bilateral four-gland neck exploration has largely been supplanted by focused single-gland excision with biochemical confirmation of a successful operation *in the operating room*. In addition, the diagnostic technology available for parathyroid localization has improved tremendously over the years, allowing elusive and ectopic abnormal parathyroid glands to be identified and localized in advance of parathyroid surgery, thus decreasing risk of unsuccessful surgical intervention and the need for reoperation. Together, preoperative imaging combined with intraoperative parathyroid hormone monitoring (IPM) allow for successful surgical outcomes and treatment of primary hyperparathyroidism at rates comparable to those of conventional bilateral neck exploration (BNE) [3–7]. While sestamibi-technetium 99m scintigraphy (sestamibi) has been considered a mainstay or essential component of focused parathyroidectomy often complemented by ultrasound (US), single-photon emission computed tomography (SPECT) imaging is a more recent advancement in preoperative parathyroid localization that may have further impact in the planning and success of targeted parathyroidectomy. 

## 2. Anatomy, Embryology, and Pathology of the Parathyroid Glands

Effective image interpretation and operative planning cannot commence without full understanding of parathyroid anatomy and its embryology. While four parathyroid glands are found in the majority of humans, cadaver studies demonstrated a 13% presence of supernumerary glands and a 3% prevalence of only three parathyroid glands [8]. At four weeks of embryologic development, the superior parathyroid glands originate from the fourth branchial pouch and undergo very little migration whereas the inferior parathyroid glands descend from the third branchial pouch to more varied locations along with the thymus. These paired superior and inferior parathyroid glands are typically symmetric in location bilaterally. In about 80% of cases, the superior and inferior parathyroid glands receive their blood supply from the inferior thyroid artery [8, 9]. 

The superior parathyroid glands are often found on the posteromedial aspect of the thyroid's superior poles approximately one centimeter above the intersection of the recurrent laryngeal nerve and inferior thyroid artery at the level of the cricoid cartilage. Since these glands undergo limited migration during embryologic development, they are rarely ectopic; however, when they do occupy ectopic domains the positions include the tracheoesophageal groove, posterior mediastinum, retroesophageal space, retropharyngeal space, and intrathyroid locations. The inferior parathyroid glands are typically located on the posterolateral surface of the inferior poles of the thyroid gland below the intersection of the recurrent laryngeal nerve and inferior thyroid artery. In about 80% of the population, the inferior parathyroid glands reside anteriorly, inferiorly, or laterally within 2 cm of the inferior pole of the thyroid gland [8, 9]. In the 20% of patients that have an ectopic inferior parathyroid gland, the most common location is within the true sheath of the thymus (15%); less frequently they are found in the intrathyroidal location (1–4%), anterior mediastinum, submandibular location, tracheoesophageal groove, retroesophageal space, and carotid sheath [8, 9]. 

The underlying pathology of primary hyperparathyroidism is most commonly a single parathyroid adenoma. This benign encapsulated neoplasm accounts for 85–96% of cases of primary hyperparathyroidism. Although most have a single affected gland, two affected glands (double adenoma) may be found in 2 to 5% of patients with primary hyperparathyroidism. Parathyroid hyperplasia is caused by an increase of parenchymal mass within all the parathyroid glands, and it accounts for 4–15% of cases. The incidence of hyperplasia increases in patients with multiple endocrine neoplasia (MEN) and non-MEN familial isolated hyperparathyroidism. Parathyroid hyperplasia is treated either by subtotal (3.5 gland) parathyroidectomy or total parathyroidectomy with autotransplantation. Parathyroid carcinoma is a very rare, indolent growing malignant neoplasm of parenchymal cells responsible for 1–5% of all primary hyperparathyroidism cases [10]. 

## 3. Primary Hyperparathyroidism

Primary hyperparathyroidism results from the overproduction of parathyroid hormone (PTH) by one or more hyperfunctioning parathyroid glands that usually causes hypercalcemia. Current widespread use of serum channel autoanalyzers has allowed for the earlier diagnosis of primary hyperparathyroidism in patients without the manifestation of clinical symptoms [11]. As the most common cause of hypercalcemia in the outpatient setting, its incidence in the general population ranges from 0.1 to 0.3%, occurring more frequently in women than men, and in those with advanced age [12–14]. Known risk factors for primary hyperparathyroidism include abnormalities of the PRAD1, MEN1, and HRPT2 genes that encode for cyclin D1, menin, and parafibromin, respectively. Radiation exposure to the neck, especially during childhood, is also associated with development of primary hyperparathyroidism [15–18]. 

The classic description of kidney “stones,” painful “bones,” abdominal “groans,” lethargic “moans,” and psychiatric “overtones” associated with primary hyperparathyroidism are now infrequently encountered in Western populations. In these parts of the world, patients with primary hyperparathyroidism present most commonly with abnormal biochemical results and not the “textbook” manifestations of primary hyperparathyroidism and hypercalcemia such as fatigue, musculoskeletal pain, polyuria, nocturia, polydipsia, constipation, heartburn, memory loss, and depression. Nonetheless, up to 80% of patients currently present with nonspecific symptoms of depression, fatigue, and lethargy, and they often are considered asymptomatic. Of note, hypertension and nephrolithiasis are the most commonly associated preoperative conditions found in patients with primary hyperparathyroidism [19, 20]. 

Biochemical diagnosis and confirmation of primary hyperparathyroidism is made by demonstrating elevated total serum or ionized calcium levels, and high intact PTH levels in the setting of normal renal function. In primary hyperparathyroidism, vitamin D metabolism is typically characterized by low plasma levels of 25-hydroxyvitamin D and high plasma levels of 1,25-dihydroxyvitamin D. Twenty-four-hour urine calcium collection is also helpful to exclude the diagnosis of benign familial hypocalciuric hypercalcemia (BFHH). A rare, autosomal dominant condition identified in patients with a family history of hypercalcemia and decreased urine calcium excretion since birth, BFHH biochemically mimics primary hyperparathyroidism by revealing elevated calcium and PTH levels, but with low levels of urinary calcium (less than 50 mg/24 hours). Similarly, the urinary calcium-to-creatinine clearance ratio in BFHH is less than 0.01 whereas in patients with primary hyperparathyroidism the ratio is greater than 0.02. However, BFHH is a benign condition that cannot be corrected by parathyroidectomy. 

Parathyroidectomy is performed in all patients with a secure diagnosis of primary hyperparathyroidism and symptoms associated with hypercalcemia. Furthermore, parathyroid surgery is indicated in asymptomatic patients with primary hyperparathyroidism that meet one or more of the criteria in the revised guidelines of the Third International Workshop on the Management of Asymptomatic Primary Hyperparathyroidism that include serum calcium >1.0 mg/dL (0.25 mmoL/liter) above normal range, creatinine clearance reduced to <60 mL/min, *T*-score <-2.5 (in lumbar spine, total hip, femoral neck, or 33% radius) and/or previous fracture fragility (use *Z*-scores in premenopausal women and men younger than 50 years), age <50, and inability to obtain continued medical surveillance [1]. 

## 4. Surgical Treatment

With the advent of improved preoperative localization techniques, increased availability of IPM, and the predominance of single-gland disease (85–96%) in patients with primary hyperparathyroidism, targeted or limited parathyroidectomy has replaced conventional BNE as the standard approach at most specialized surgical centers worldwide [3–7]. Advantages of targeted parathyroidectomy include improved cosmetic results with smaller incisions, decreased pain, shorter operative time, ambulatory surgery, decreased hospitalization, quick postoperative recovery, less frequent injury to the recurrent laryngeal nerve, decreased postoperative hypocalcemia, and comparable success rates to conventional BNE [4–7]. 

Many techniques of focused parathyroidectomy have been described that incorporate and share the common aspects and principles of minimally invasive surgery, such as less dissection, decreased operative time, less morbidity, and comparable reported operative success to BNE ranging from 97% to 99%. For targeted parathyroidectomy to be successful, precise preoperative localization is essential. These focused procedures are performed in patients with a single parathyroid adenoma localized by preoperative sestamibi and/or US through a central or lateral incision measuring from 2 to 4 cm. Only the abnormal parathyroid gland is identified and excised. IPM is used by most endocrine surgeons to confirm that no additional hypersecreting parathyroid tissue remains. During this operation, blood is drawn to measure parathyroid hormone levels at baseline and after excision of a hyperfunctioning parathyroid gland. When IPM levels decrease by >50% 10 minutes after gland excision, the limited operation is completed. If a >50% drop does not occur, either a double adenoma or 4-gland hyperplasia is present and bilateral neck exploration is then performed. Under general or local anesthesia, targeted parathyroidectomy can be offered to most patients in the outpatient setting. Patients with known multiglandular disease (MGD) preoperatively are not offered this focused approach. 

## 5. Preoperative Parathyroid Localization

Preoperative localization of hyperfunctioning parathyroid tissue is an essential component of focused parathyroidectomy. Parathyroid localization has improved with a variety of familiar imaging techniques including sestamibi scintigraphy, ultrasonography, and four-dimensional computed tomography, which has the added dimension of changes in perfusion of contrast over time compared to regular 3-D CT. Comparisons of these different imaging modalities have shown the superiority of scintigraphy for preoperative parathyroid localization. First reported in 1989, technetium (99 mTc) sestamibi is used for parathyroid scintigraphy as a radiotracer injected intravenously where the patient's neck is later imaged with a gamma camera [21]. 99 mTc sestamibi consists of lipophilic cationic molecules. After intravenous injection, the molecules are distributed throughout the circulatory system, into cells by passive diffusion, and become concentrated intracellularly in mitochondria. Approximately two hours after injection, thyroid cells lose significant sestamibi uptake whereas abnormal parathyroid gland oxyphil cells retain the marker in high mitochondrial concentrations that assist in parathyroid adenoma localization. Sestamibi has been regarded as the single best imaging modality for parathyroid adenoma identification over ultrasonography, CT, and magnetic resonance imaging [22–24]. Notwithstanding, this technology has its limitations. The radiotracer provides only two-dimensional, planar images. Sestamibi can localize 80% to 90% of single abnormal parathyroid glands, but it is less sensitive in the diagnosis of MGD [25–27]. Thyroid nodules or lymph nodes can also mimic abnormal parathyroid glands and cause false-positive results on sestamibi scans [28]. 

**Figure 1:**
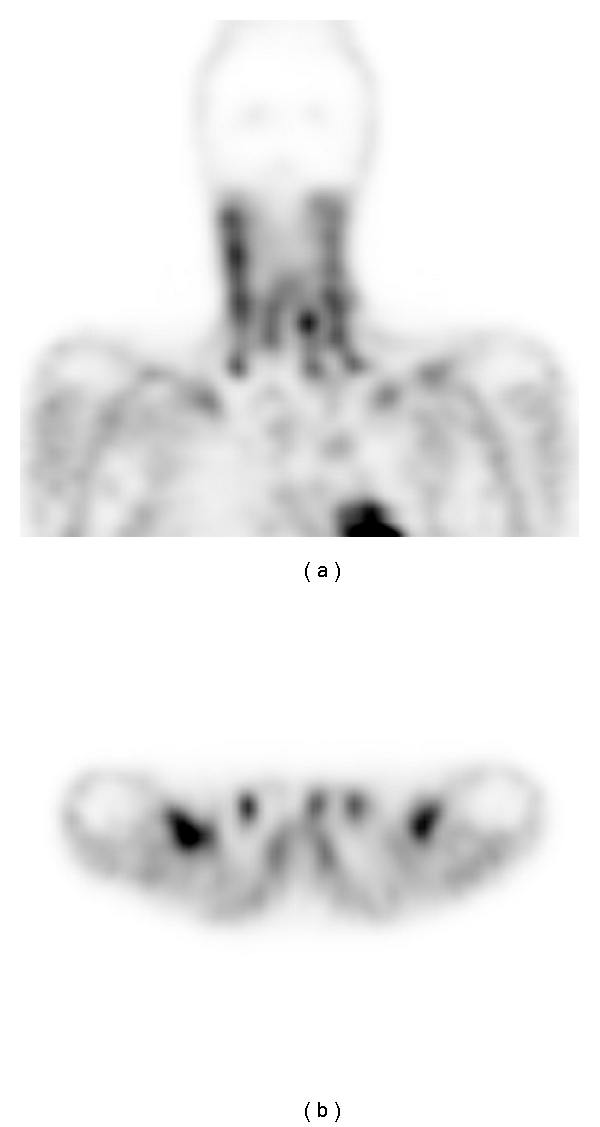
Coronal (a) and transverse (b) tomograms from a delayed phase SPECT in a patient with a left superior parathyroid adenoma confirmed at surgery.

**Figure 2:**
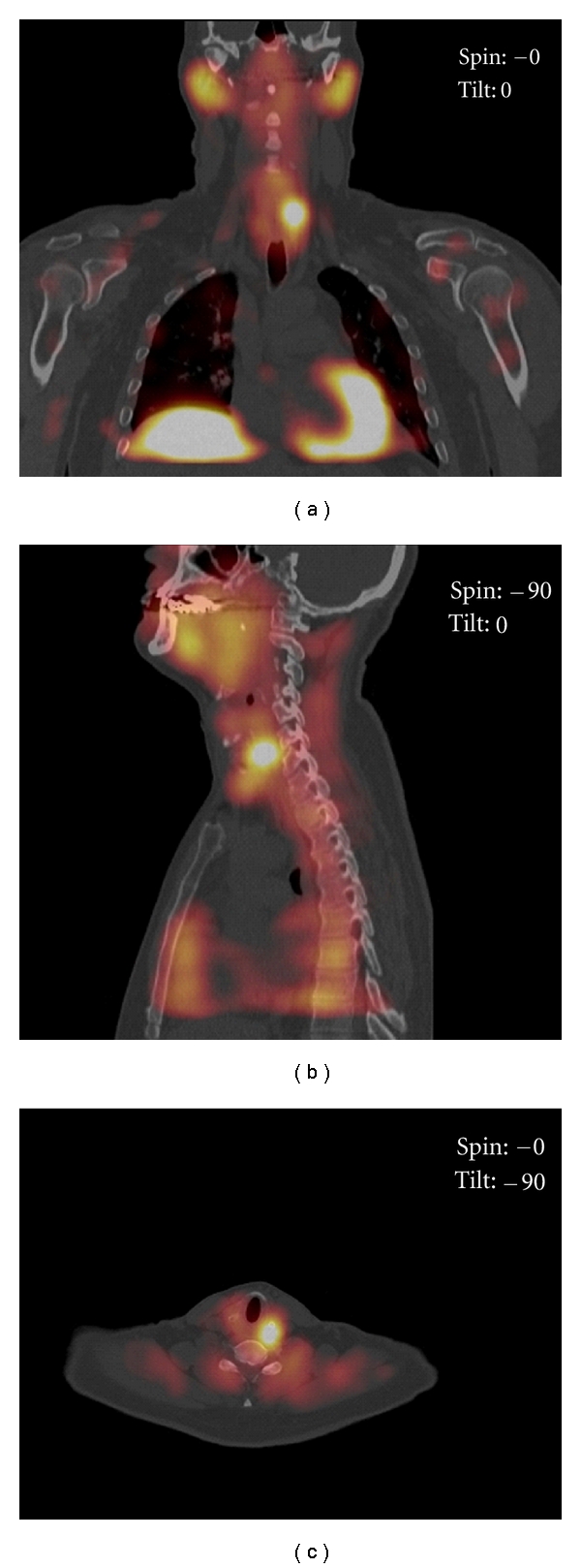
Left superior parathyroid adenoma with delayed washout. Delayed phase (a) coronal, (b) sagittal, and (c) transverse fused SPECT/CT tomograms show a left superior parathyroid adenoma with a posterior location at the upper pole of the left thyroid lobe.

SPECT is currently used with increased frequency due to the three-dimensional information it provides with an improved sensitivity for the detection and localization of hyperfunctioning parathyroid glands (Figure 1). SPECT measures gamma radiation and obtains multiple angle images of the neck and mediastinum and then merges all cuts to reconstruct a three-dimensional image. The advanced contrast resolution of SPECT is the primary reason for its superiority over other imaging methods. Multiple studies have established the role and advantages of SPECT in the improvement of parathyroid localization. In one of the largest studies looking at SPECT in over 550 patients with primary hyperparathyroidism, radiologists developed a scoring system to predict parathyroid pathology based on intensity and pattern of sestamibi uptake. Patients with a SPECT image reading as “probable” or “definite” for parathyroid adenoma had a positive predictive value (PPV) of >94% for adenoma presence and correct laterality localization upon review of surgical findings. Patients with negative scans, however, had a higher rate of operative failure. Multiglandular disease was still not well predicted [29]. Investigators have also come to appreciate that using a hybrid SPECT/CT scan can further enhance localization by providing better resolution of surrounding structures. The fusion of CT with SPECT images allows for the combined anatomic information from CT and the physiologic three-dimensional information from SPECT (Figure 2). This SPECT/CT combination has the added benefit of a more precise anatomic localization of ectopic and mediastinal parathyroid adenomas. 

In a direct comparison study of sestamibi scintigraphy, SPECT, and SPECT/CT in patients with severe primary hyperparathyroidism, patients had either multinodular goiters with unclear gland identification by US, normal thyroid glands with negative parathyroid localization by US, or ectopic glands demonstrated by planar parathyroid scintigraphy [30]. All patients underwent the three imaging modalities. Results revealed that double-phase planar scintigraphy only showed the presence of 14 probable hyperfunctioning parathyroid glands (12 in eutopic, 2 in ectopic locations) of 23 lesions localized at surgery (61%) whereas SPECT showed the presence of 23 probable adenomas (9 eutopic, 14 ectopic), but only correctly identified the location of 14 out of 23 lesions (61%) with extreme precision. SPECT/CT, however, identified the presence and correct location of 100% (all 23) of the lesions, and it was the only modality that identified retrotracheal parathyroid glands in three different patients. 

An evaluation of planar imaging, SPECT, and SPECT/CT scintigraphy was performed on 98 patients with primary hyperparathyroidism caused by single adenoma with no previous neck surgery [31]. Each patient was subjected to planar imaging, SPECT, and SPECT/CT at 15 minutes and two hours after sestamibi injection. Surgical location served as the standard. Early SPECT/CT in combination with any delayed (two-hour) imaging method was significantly superior to any single- or dual-phase planar or SPECT study in regards to sensitivity and positive predictive value for abnormal parathyroid localization. Parathyroid localization had a sensitivity of 34% for single-phase early planar images, 45% for single-phase delayed planar images, 57% for dual-phase planar, 54% for single-phase early SPECT, 54% for single-phase delayed SPECT, and 62% for dual-phase SPECT that increased to 73% sensitivity for dual-phase studies with early SPECT/CT. Specificity and negative predictive values remained constant across all modalities. Localization with dual-phase acquisition was also found to be more accurate than with single-phase 99 mTc-sestamibi scintigraphy for planar imaging, SPECT, and SPECT/CT. This study concluded that dual-phase imaging with early SPECT/CT should be incorporated into routine preoperative planning for all patients with primary hyperparathyroidism. 

Other studies have reported similar sensitivities, but also impressive specificity for SPECT/CT in localizing parathyroid adenomas. In one study comparing SPECT and SPECT/CT in 61 patients with primary hyperparathyroidism, although the sensitivities of SPECT (71%) and SPECT/CT (70%) were similar (*P* = .779), the specificity of SPECT/CT (96%) was significantly greater than that of SPECT alone (48%; *P* = .006) [32]. In another report of 116 patients with single-gland disease, sensitivity for SPECT/CT was 88%, for CT 70%, and for SPECT 59%. Specificity for SPECT/CT was 99%, for SPECT 95%, and for CT 94% [33]. Both studies concluded that SPECT/CT fusion was superior to the individual CT or SPECT images alone in localizing parathyroid adenomas.

In a study of 28 patients undergoing reoperative surgery for a “missed” parathyroid gland, SPECT/CT was able to predict the exact position of the abnormal gland in 86% of the patients whereas SPECT was successful in only 43% of cases (*P* < .004). SPECT/CT detected all three pathologic glands in ectopic positions [34]. Further reports supported that the additional information provided by hybrid SPECT/CT imaging often proved to be advantageous in the detection and localization of ectopic parathyroid adenomas [35]. Another population in which SPECT/CT might prove beneficial is patients with multinodular goiter and primary hyperparathyroidism. In a study of 33 patients with these two diagnoses, all study individuals underwent preoperative sestamibi planar scintigraphy and SPECT (18 patients) or SPECT/CT (15 patients) after cervical ultrasound [36]. SPECT/CT showed higher sensitivity than SPECT (87.5% versus 55.6%; *P* = .0001) and higher PPV (87.5% versus 62.5%; *P* = .0022) for correctly identifying the neck quadrant affected by primary hyperparathyroidism; the specificity trended toward SPECT/CT over SPECT, but was not significant (95.5% and 88.5%, resp.). The report concluded that SPECT/CT is superior to SPECT for preoperative imaging of patients with both primary hyperparathyroidism and multinodular goiter, and should be a routine part of this population's preoperative workup. 

More studies concur that SPECT/CT offers enhanced ability to localize abnormal parathyroid glands above planar scintigraphy or SPECT alone. In investigating the role of SPECT/CT in the general oncologic patients, the functional and anatomic imaging obtained from SPECT and CT has been shown to be synergistic versus complementary, and can significantly contribute to more accurate localization of parathyroid and other cancers [37]. While the SPECT/CT can be technically challenging to perform and potentially increase patient radiation doses, the benefits of its improved sensitivity and specificity can decrease need for future confirmatory imaging studies [33, 35]. 

## 6. Conclusion

Primary hyperparathyroidism is caused by a single parathyroid gland in up to 96% of cases. Focused parathyroidectomy is the preferred treatment of choice for this condition, and scintigraphy is a principal method used for preoperative parathyroid gland localization. SPECT is more useful than planar imaging since it provides additional information about the superior/inferior and anterior/posterior location of abnormal parathyroid glands. Fusion of CT with SPECT has the added benefit of providing not only useful anatomic information but functional assessment as well. For patients with ectopic glands or patients facing reoperation, SPECT/CT is particularly helpful for preoperative localization and may become the preferred method of parathyroid detection and localization in such clinical scenarios. Nevertheless, the long-term clinical and economic benefits of SPECT and SPECT/CT, although promising, remain to be determined. 

## References

[1] Bilezikian JP, Khan AA, Potts JT (2009). Guidelines for the management of asymptomatic primary hyperparathyroidism: summary statement from the third international workshop. *Journal of Clinical Endocrinology and Metabolism*.

[2] Mandl F (1926). Therapeutischer versuch bein einem falls von otitis fibrosa generalisata mittles. Exstirpation eines epithelkorperchentumors. *Wien Klin Wochenschr Zentral*.

[3] Sackett WR, Barraclough B, Reeve TS, Delbridge LW (2002). Worldwide trends in the surgical treatment of primary hyperparathyroidism in the era of minimally invasive parathyroidectomy. *Archives of Surgery*.

[4] Udelsman R (2002). Six hundred fifty-six consecutive explorations for primary hyperparathyroidism. *Annals of Surgery*.

[5] Westerdahl J, Lindblom P, Bergenfelz A (2002). Measurement of intraoperative parathyroid hormone predicts long-term operative success. *Archives of Surgery*.

[6] Irvin GL, Carneiro DM, Solorzano CC (2004). Progress in the operative management of sporadic primary hyperparathyroidism over 34 years. *Annals of Surgery*.

[7] Grant CS, Thompson G, Farley D (2005). Primary hyperparathyroidism surgical management since the introduction of minimally invasive parathyroidectorny: Mayo Clinic experience. *Archives of Surgery*.

[8] Akerstrom G, Malmaeus J, Bergstrom R (1984). Surgical anatomy of human parathyroid glands. *Surgery*.

[9] Thompson NW, Eckhauser FE, Harness JK (1982). The anatomy of primary hyperparathyroidism. *Surgery*.

[10] Givi B, Shah JP (2010). Parathyroid carcinoma. *Clinical Oncology*.

[11] Wermers RA, Khosla S, Atkinson EJ (2006). Incidence of primary hyperparathyroidism in Rochester, Minnesota, 1993–2001: an update on the changing epidemiology of the disease. *Journal of Bone and Mineral Research*.

[12] Heath H, Hodgson SF, Kennedy MA (1980). Primary hyperparathyroidism. Incidence, morbidity, and potential economic impact in a community. *The New England Journal of Medicine*.

[13] Chen H, Parkerson S, Udelsman R (1998). Parathyroidectomy in the elderly: do the benefits outweigh the risks?. *World Journal of Surgery*.

[14] Uden P, Chan A, Duh QY, Siperstein A, Clark OH, Irvin GL (1992). Primary hyperparathyroidism in younger and older patients: symptoms and outcome of surgery. *World Journal of Surgery*.

[15] Arnold A, Kim HG, Gaz RD (1989). Molecular cloning and chromosomal mapping of DNA rearranged with the parathyroid hormone gene in a parathyroid adenoma. *Journal of Clinical Investigation*.

[16] Heppner C, Kester MB, Agarwal SK (1997). Somatic mutation of the MEN 1 gene in parathyroid tumours. *Nature Genetics*.

[17] Carpten JD, Robbins CM, Villablanca A (2002). HRPT2, encoding parafibromin, is mutated in hyperparathyroidism-jaw tumor syndrome. *Nature Genetics*.

[18] Christensson T (1978). Hyperparathyroidism and radiation therapy. *Annals of Internal Medicine*.

[19] Eigelberger MS, Cheah WK, Ituarte PH, Streja L, Duh QY, Clark OH (2004). The NIH criteria for parathyroidectomy in asymptomatic primary hyperparathyroidism: are they too limited?. *Annals of Surgery*.

[20] Bilezikian JP, Potts JP (2002). Asymptomatic primary hyperparathyroidism: new issues and new questions. *Journal of Bone and Mineral Research*.

[21] Coakley AJ, Kettle AG, Wells CP (1989). 99Tcm sestamibi: a new agent for parathyroid imaging. *Nuclear Medicine Communications*.

[22] Ishibashi M, Nishida H, Hiromatsu Y (1998). Comparison of technetium-99m-MIBI, technetium-99 m-tetrofosmin, ultrasound and MRI for localization of abnormal parathyroid glands. *Journal of Nuclear Medicine*.

[23] Geatti O, Shapiro B, Orsolon PG (1994). Localization of parathyroid enlargement: experience with technetium-99 m methoxyisobutylisonitrile and thallium-201 scintigraphy, ultrasonography and computed tomography. *European Journal of Nuclear Medicine*.

[24] Peeler BB, Martin WH, Sandler MP, Goldstein RE (1997). Sestamibi parathyroid scanning and preoperative localization studies for patients with recurrent/persistent hyperparathyroidism or significant comorbid conditions: development of an optimal localization strategy. *American Surgeon*.

[25] Chiu B, Sturgeon C, Angelos P (2006). What is the link between nonlocalizing sestamibi scans, multigland disease, and persistent hypercalcemia? A study of 401 consecutive patients undergoing parathyroidectomy. *Surgery*.

[26] Carniero-Pla DM, Solorzano CC, Irvin GL (2006). Consequences of targeted parathyroidectomy guide by localizing studies without intraoperative parathyroid hormone monitoring. *Journal of the American College of Surgeons*.

[27] Yip L, Pryma DA, Yim JH, Virji MA, Carty SE, Ogilvie JB (2008). Can a lightbulb sestamibi SPECT accurately predict single-gland disease in sporadic primary hyperparathyroidism?. *World Journal of Surgery*.

[28] Palestro CJ, Tomas MB, Tronco GG (2005). Radionuclide imaging of the parathyroid glands. *Seminars in Nuclear Medicine*.

[29] Yip L, Pryma DA, Yim J, Carty SE, Ogilvie JB (2009). Sestamibi SPECT intensity scoring system in sporadic primary hyperparathyroidism. *World Journal of Surgery*.

[30] Serra A, Bolasco P, Satta L, Nicolosi A, Uccheddu A, Piga M (2006). Role of SPECT/CT in the preoperative assessment of hyperparathyroid patients. *Radiologia Medica*.

[31] Lavely WC, Goetze S, Friedman KP (2007). Comparison of SPECT/CT, SPECT, and planar imaging with single- and dual-phase ^99m^Tc-sestamibi parathyroid scintigraphy. *Journal of Nuclear Medicine*.

[32] Neumann DR, Obuchowski NA, Difilippo FP (2008). Preoperative ^123^I/^99m^Tc-sestamibi subtraction SPECT and SPECT/CT in primary hyperparathyroidism. *Journal of Nuclear Medicine*.

[33] Prommegger R, Wimmer G, Profanter C (2009). Virtual neck exploration: a new method for localizing abnormal parathyroid glands. *Annals of Surgery*.

[34] Wimmer G, Bale R, Kovacs P (2008). Virtual neck exploration in patients with hyperparathyroidism and former cervical operations. *Langenbeck’s Archives of Surgery*.

[35] Levine DS, Belzberg AS, Wiseman SM (2009). Hybrid SPECT/CT imaging for primary hyperparathyroidism: case reports and pictorial review. *Clinical Nuclear Medicine*.

[36] Pata G, Casella C, Besuzio S (2010). Clinical appraisal of 99m technetium-sestamibi SPECT/CT compared to conventional SPECT in patients with primary hyperparathyroidism and concomitant nodular goiter. *Thyroid*.

[37] Núñez R (2010). Acquisition parameters for oncologic imaging with a new SPECT/multislice CT scanner. *Molecular Imaging and Biology*.

